# A Performance Study of Mobility Speed Effects on Vehicle Following Control via V2V MIMO Communications

**DOI:** 10.3390/s25041193

**Published:** 2025-02-15

**Authors:** Jerawat Sopajarn, Apidet Booranawong, Surachate Chumpol, Nattha Jindapetch, Okuyama Yuichi, Hiroshi Saito

**Affiliations:** 1 Department of Electrical and Biomedical Engineering, Faculty of Engineering, Prince of Songkla University, Hat Yai 90110, Songkhla, Thailand; 6110130005@email.psu.ac.th (J.S.); nattha.s@psu.ac.th (N.J.); 2 Toyota Tsusho NEXTY Electronics (Thailand) Company Ltd., Bangkok 10330, Thailand; surachate@th.nexty-ele.com; 3 Division of Computer Engineering, The University of Aizu, Aizu-Wakamatsu 965-8580, Japan; okuyama@u-aizu.ac.jp

**Keywords:** mobility speed, vehicle-following control, V2V communications, MIMO

## Abstract

Vehicle-to-vehicle (V2V) communications are important for intelligent transportation system (ITS) development for driving safety, traffic efficiency, and the development of autonomous vehicles. V2V communication channels, environments, mobility patterns, and mobility speed significantly affect the accuracy of autonomous vehicle control. In this paper, we propose a versatile system-level framework that can be used for investigation, experimentation, and verification to expedite the development of autonomous vehicles. Once vehicle functionality, communication channels, and driving scenarios were modelled, experiments with different mobility speeds and communication channels were set up to measure the communication quality and the effects on the vehicle’s following control. In our experiment, the leader vehicle was set to travel through a high-building environment with a constant speed of 36 km/h and suddenly changed lanes in front of the follower vehicle. The speed of the follower vehicle ranged from 40 km/h to 80 km/h. The experimental results show that the quality of single-input and single-output (SISO) communication is less efficient than multiple-input and multiple-output (MIMO) communication. The quality of SISO communication between vehicles with a speed difference of 4 km/h (leader 36 km/h and follower 40 km/h) had a link quality worse than 0.85, which caused unstable control in the follower vehicle speed. However, it was also found that if the speed of the follower vehicle increased to 80 km/h, the link quality of SISO communication was better, close to 0.95, due to the decreased distance between the vehicles, resulting in better control. Moreover, it was found that the impact of SISO communication can be overcome by using the MIMO communication technique and selecting the best input signal at each time. MIMO communication has less signal loss, allowing the follower vehicle to make correct decisions throughout the movement.

## 1. Introduction

Vehicle-to-vehicle (V2V) communications are essential for intelligent transportation system (ITS) development, including driving safety, traffic efficiency, and the advancement of self-driving vehicles [1,2]. Information from other cars, such as position, speed, and braking status, is important for driving decisions. The main problem with V2V communications is the environmental impacts and, more importantly, the speed of vehicle mobility. Therefore, it is necessary to further study the impact of speed on the efficiency of autonomous vehicle control.

The ITS protocol stack ITS-G5/IEEE802.11p allows V2V communications to send road traffic status and emergency notifications to nearby vehicles. Vehicle density and speed variations are important to the effectiveness of V2V communications. The packet reception ratio (PRR) between transmitter and receiver and the end-to-end (E2E) delay are common performance metrics to evaluate communication reliability and latency. According to a literature survey, a messaging strategy based on ITS-G5 was developed to send urgent notifications to road users, and the evaluated performance at a chosen site reported that the PRR value for V2V transmissions varied when the number of cars and their speeds increased [3]. A vehicular ad hoc network (VANET), an ITS-G5 and 5G test network, with drone-assisted communication, was proposed to improve the communication range [4]. More measurements in real environments were required in high vehicle mobility and complex traffic environments.

In recent years, antennas and radio propagation technologies have been developed to enhance V2V communications. The propagation loss characteristics and an antenna configuration suitable for V2V application have been studied [5]. The evaluation of a model for a cognitive V2V communication system with two active user cooperation revealed that, as the power from the primary user increased, the V2V user failed to achieve the standard bit error rate (BER) requirement [6]. Preliminary results of genetic algorithms (GAs) to learn the beam shapes of an antenna array according to the surrounding environment could improve V2V communications in urban intersections [7]. Also, a federated learning approach for switched beam antennas could significantly reduce the latency and throughput overhead in V2V networks [8].

Because V2V communication channels are critical in autonomous vehicles, the statistical properties of different taps in different scenarios of a three-dimensional (3D) geometry-based stochastic model (GBSM) for V2V multiple-input and multiple-output (MIMO) wideband channels were studied. The results showed that the vehicle speed in urban canyons was the lowest, and the Doppler frequency caused by motion was the smallest [9]. A set of MATLAB-based simulation results for vehicle-to-everything (V2X) communication indicated that data exchange between high-speed vehicles lowers the quality of transmission [10]. The OpenStreetMap (OSM) incorporated with the SUMO simulator and network simulator version 2 (NS2) also reported that higher-speed vehicles suffered from decreased data delivery efficiency compared to lower-speed vehicles [11]. According to [12,13], different VANET protocols performed differently depending on the mobility speed and network density. The link lifetime between vehicles travelling in the same and opposite directions was compared in [14,15]. However, these studies did not consider the effect of communication channels on autonomous vehicle control.

An investigation of the performance impact of mobility speed differences among the nodes in a platoon of vehicles revealed a major performance shift between the furthest distance (FD) and the link-quality-based bi-directional stable communication (BDSC) scheme [16]. Finally, a model-based design (MBD) framework was developed to model the vehicle-following control, autonomous driving scenarios, and single-input and multiple-output (SIMO) communication channels [17]. However, the mobility speed’s effect on autonomous vehicle control in different channel models needs to be further investigated.

Table 1 summarizes not only the above-mentioned literature review but also a review of the development of V2V communication systems reported in the literature in comparison to our proposed system and its functionality. The novelty and contributions of this work are as follows:

- In this paper, we present a versatile system-level framework used for the development of autonomous vehicle systems. We particularly focus on the mobility speed’s effect on the performance of vehicle-following control via V2V SISO and MIMO communications.
- Modelled vehicle functions, communication channels, and driving scenarios were tested with various vehicle speeds and channels to assess the communication quality and its impact on vehicle control.
- Our major findings indicate that the SISO and MIMO communication techniques between vehicles with speed differences significantly affected the communication link quality and the efficiency of vehicle control. The impact of SISO communications can be overcome by using MIMO communications and selecting the best input signal. MIMO communication has less signal loss, allowing the vehicle to make correct decisions while moving.

This paper is organized as follows: Section 2 describes the vehicle control system via the V2V communication introduced in this work, including a modelling framework, vehicle control systems, vehicle communication, and mobility speed factors. Section 3 provides the simulation setup, where the vehicles’ movement situations and speeds in each test scenario are detailed. The results and discussion are in Section 4. Finally, we conclude the paper in Section 5.

## 2. Vehicle Control System via V2V Communications

To study the effects of mobility speed on vehicles controlled via V2V MIMO communications, we first introduce our system presented in this work, which includes a modelling framework for V2V communication systems, vehicle control systems, vehicle communication, and mobility speed factors.

### 2.1. Modelling Framework

**Figure 1:**
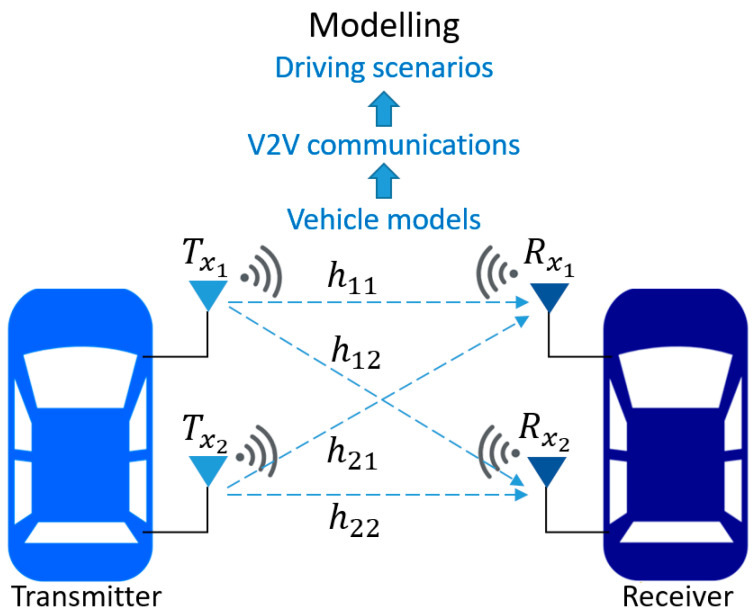
Modelling framework for V2V communication systems.

Currently, the process of controlling autonomous vehicles adopts a communication system between vehicles to enable vehicles to communicate with each other and share information for decision-making and safety. The essential factor for controlling intelligent vehicles is accuracy. Therefore, it is necessary to have a versatile system-level framework that can be used for investigation, experimentation, and verification to expedite the development of autonomous vehicles. In this work, the proposed framework uses a model-based design (MBD) methodology for modelling vehicle functionality, V2V communications, and driving scenarios, as shown in Figure 1.

- The vehicle model: vehicle models (i.e., type of car) and vehicle functions, such as sensor data readings, global positioning systems (GPSs), autonomous emergency braking controls, velocity decision controls, path-following controls, vehicle dynamics, and so on, can be designed and tested.
- V2V communications: SISO and MIMO communication techniques employing wireless signals between vehicles can be modelled. Furthermore, environmental conditions, such as urban areas, high-building environments, highways, and so on, which have a direct impact on wireless communication channels between vehicles, can be identified.
- Driving scenarios: driving scenarios or test cases can be designed and assessed, where features such as the number of vehicles, vehicle velocity, and mobility direction and patterns can be set.

### 2.2. Vehicle Control System

**Figure 2:**
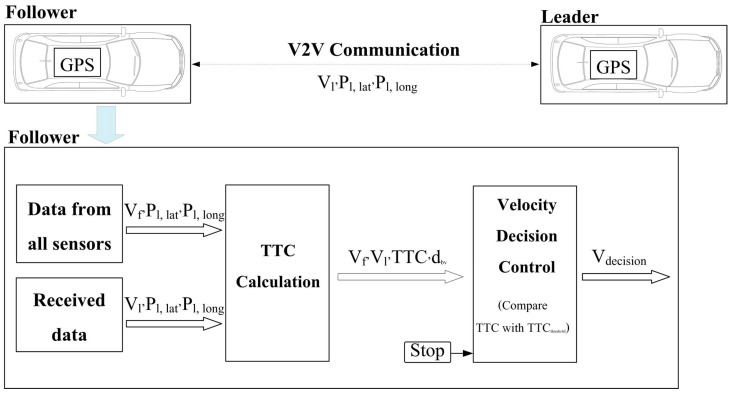
The vehicle-following model [17]; V_l_, V_f_, P_l,lat_, P_l,long_, TTC, d_bv_, and V_decision_ refer to the leader velocity, follower velocity, leader position (latitude), leader position (longitude), time-to-collision, distance between vehicles, and decision velocity, respectively.

According to the above modelling framework, the vehicle-following model is demonstrated in Figure 2. In this model, the leader and the follower vehicles are connected together via V2V communications. The functions of the follower vehicle include data reading from all sensors employed in the vehicle (i.e., GPS, velocity sensor, radar, camera, etc.), data receiving, time-to-collision (TTC) calculations (i.e., the ratio of the distance between vehicles and the difference in velocities between vehicles, as further described in Appendix A), velocity decision control, path-following control, and vehicle dynamics, respectively. This vehicle-following model was designed and implemented in MATLAB/Simulink, and more details can be found in [17].

**Figure 3:**
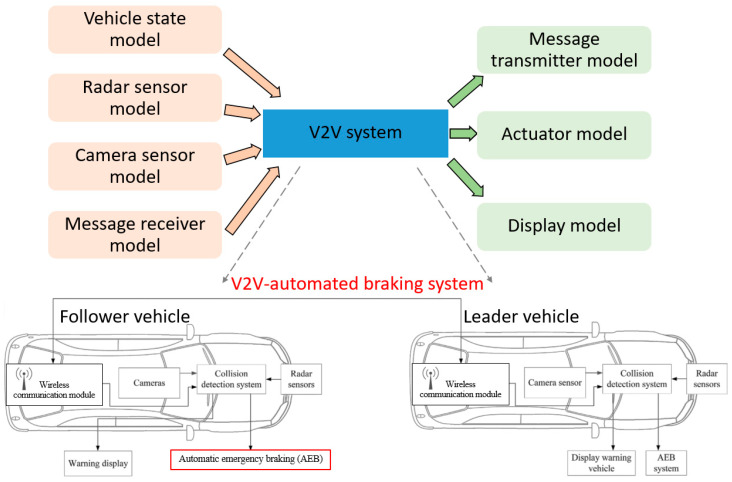
The V2V-automated braking system in the vehicle model.

In the vehicle-following model, one of the adaptive cruise control system’s main functions is designed to assist in controlling vehicle speeds in order to improve road traffic efficiency, minimize accident rates, and reduce vehicle energy consumption. In autonomous mode, the vehicle-following system coordinates the driving maneuvers of the leader and the follower vehicle. To demonstrate the potential of the approach, an automated brake model is shown as follows:

Figure 3 illustrates a V2V simulation scenario with an autonomous braking system. The middle block represents the V2V automated braking system, while the two side blocks are models for sensors, data reception, data transmission, actuation, and data display. The V2V-automated braking model uses signals received from sensors (i.e., vehicle state model, radar sensor model, camera sensor model) and other vehicles (i.e., message receiver model) to detect possible collisions with other vehicles and make appropriate decisions to avoid collisions. The outputs of the model are the actuator model to control the vehicle speed and the data display model to display the data. The message transmitter model is also used to notify other vehicles. We note that, in this experiment, the type and number of sensors used for the V2V-automated braking system can be flexibly configured. Table 2 also illustrates the input information for the V2V-automated braking system, where velocity, acceleration, throttle state, brake state, steering angle, and target ID are taken into consideration.

**Figure 4:**
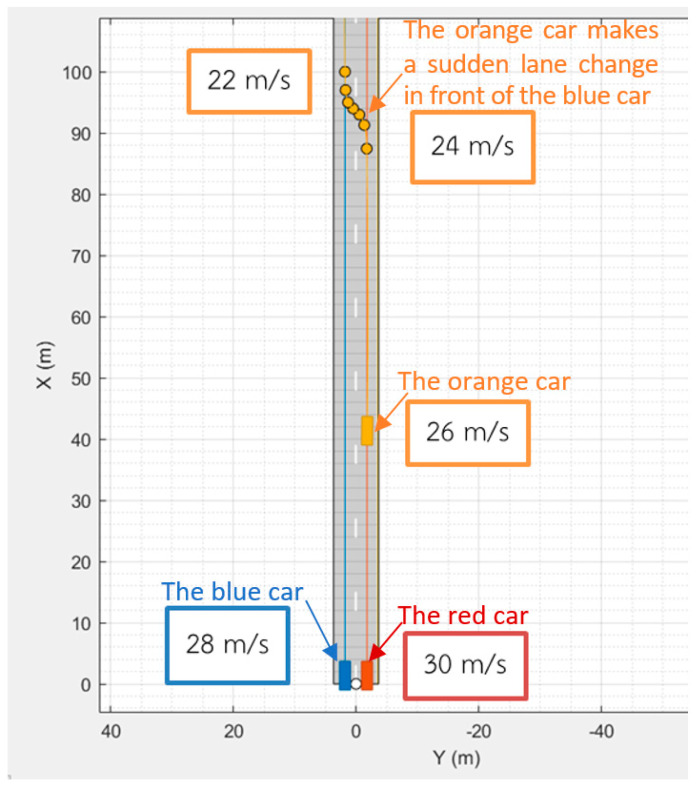
Simulation example of a vehicle-following scenario; the orange car makes a sudden lane change in front of the blue car. The blue car will make a decision and action based on the V2V-automated braking system.

The simulation scenario for V2V-automated braking is illustrated here. In the V2V-automated braking simulation process, accidents are possible when there is a sudden lane change. Therefore, vehicles need to have sensors and communication systems to avoid collisions. Figure 4 shows the example scenario used for the V2V-automated braking simulation. This scenario consists of three vehicles, where each vehicle has the following state variables: position, speed, and acceleration. The speeds of the vehicles are assigned as follows: The blue car drives at a constant speed of 28 m/s along the road, and the red car drives at 30 m/s. The orange car drives at 26 m/s, 24 m/s, and 22 m/s and makes a sudden lane change in front of the blue car. In this case, the blue car will make the decision and action based on the V2V-automated braking system.

To show the potential of this system, the experimental setup in Section 3 related to the mobility speed’s effects on vehicle-following control via V2V communications will be described. The results and discussion related to this issue will be shown in Section 4.

### 2.3. V2V Communications

**Figure 5:**
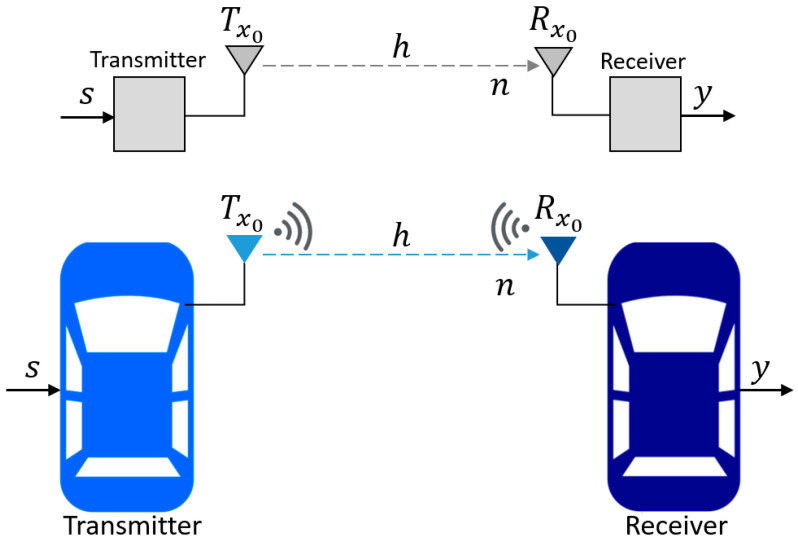
SISO communications.

Since vehicle-following control via V2V communications for autonomous vehicles in urban environments is considered in this work, this section summarizes SISO and MIMO communications. SISO communications are shown in Figure 5. SISO is a common format for wireless communication systems due to its simplicity. The SISO system consists of a single transmitter (Tx0) and one receiver (Rx0).

The output signal of SISO communication can be expressed by (1). Here, $y_{i}$ is the output signal or the received signal, s is the input signal or the transmitted signal, $h_{i}$ is the channel response, and $n_{i}$ is the noise of the communication system.

(1) $$y_{i}=h_{i}*s+n_{i}$$

**Figure 6:**
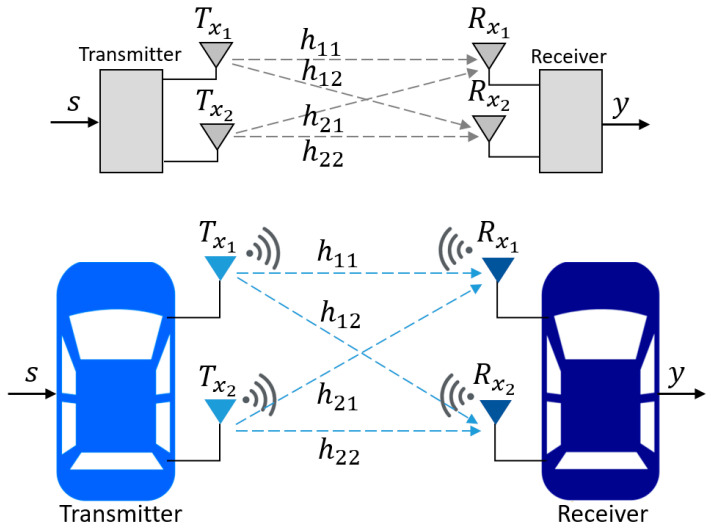
MIMO communications.

Because multipath fading effects degrade the radio signal quality of SISO communications in certain environments, such as those with tall buildings, trees, bridges, and other obstacles, essential parameters of autonomous vehicles cannot be accurately measured. Instead, a more reliable communication technology, known as MIMO, was used. MIMO communication consists of a transmitter and a receiver with many antennas (two antennas each, in this study), as seen in Figure 6. The output signal Y can be determined by (2). Here, s is the input signal, H refers to the channel matrix, and N is the noise in all the communication channels. For one transmitter with two antennas and one receiver with two antennas, Y, H, and N are determined in (3) to (5).

Note: $Y\to \left[\begin{array}{c} y_{1}\\ y_{2} \end{array}\right]$, $H\to \left(\begin{array}{cc} h_{1,1} & h_{1,2}\\ h_{2,1} & h_{2,2} \end{array}\right)$, $S\to \left(\begin{array}{c} x_{1}\\ x_{2} \end{array}\right)$, and $N\to \left(\begin{array}{c} n_{1}\\ n_{2} \end{array}\right)$.

(2) Y=Hs+N

(3) $$\left[\begin{array}{c} y_{1}\\ y_{2} \end{array}\right]=\left(\begin{array}{cc} h_{1,1} & h_{1,2}\\ h_{2,1} & h_{2,2} \end{array}\right)\left(\begin{array}{c} x_{1}\\ x_{2} \end{array}\right)+\left(\begin{array}{c} n_{1}\\ n_{2} \end{array}\right)$$

(4) $$y_{1}=h_{1,1}x_{1}+h_{1,2}x_{2}+n_{1}=\left[\begin{array}{cc} h_{1,1} & h_{1,2} \end{array}\right]\left[\begin{array}{c} x_{1}\\ x_{2} \end{array}\right]+n_{1}$$

(5) $$y_{2}=h_{2,1}x_{1}+h_{2,2}x_{2}+n_{2}=\left[\begin{array}{cc} h_{2,1} & h_{2,2} \end{array}\right]\left[\begin{array}{c} x_{1}\\ x_{2} \end{array}\right]+n_{2}$$

In this work, the leader vehicle is the transmitter with two antennas, while the follower vehicle is the receiver with two antennas. The redundant antenna increases the reception diversity, wherein the same data is received across separate fading channels in order to mitigate the effects of fading.

### 2.4. Mobility Speed Factor

According to the pioneer study in [16], the maximum link lifetime duration (i.e., $T_{Conn}$) in which two vehicles in a pair can maintain their connectivity with each other can be determined using (6). A fixed transmission range is R, and ∆v refers to the speed difference between two vehicles, where the leader vehicle has a higher mobility speed $v_{1}$ compared to the follower vehicle $v_{2}$. The equation is applied when both vehicles maintain constant mobility speeds.

$$T_{Conn}=\left(\frac{R}{∆v}\right)\times 2$$

(6) $$∆v=v_{1}-v_{2}$$

For example, if a fixed communication range R is 300 m and $v_{1}$ and $v_{2}$ are 101 km/h and 96 km/h, respectively, $T_{Conn}$ is equal to 432 s. Note that in another pair, in which $v_{1}=86$ km/h and $v_{2}=81$ km/h, the link lifetime duration would be the same. By considering this equation, if the speed difference ∆v is small, the maximum link lifetime duration $T_{Conn}$ will be large. In contrast, the bigger the ∆v, the shorter the $T_{Conn}$. For example, if $v_{1}=135$ km/h and $v_{2}=75$ km/h, ∆v will be 60 km/h and $T_{Conn}$ will be 36 s, which is a short link lifetime duration.

This information reflects how small and large mobility speed differences affect the link lifespan duration of two vehicles going in the same direction. We will provide a discussion related to this issue in Section 4.

## 3. Simulation Setup

Due to the necessity of studying the effect of speed on autonomous vehicle control in different channels, the experiment was designed using the modelling framework for the V2V communication system proposed in Figure 1 to analyze the mobility speed, scenario, and autonomous control system. In this section, an experimental setup is described, including IEEE802.11p parameters, vehicle movement scenarios, mobility speeds for testing, and SISO and MIMO evaluations.

Since the IEEE 802.11p is an approved amendment to the IEEE 802.11 standard that includes wireless access in a vehicular communication system [38], the most important parameters related to this standard are demonstrated in Table 3. A comparison of the IEEE802.11a and 802.11p parameters in the physical layer is listed. Note that the IEEE 802.11p adapts the IEEE 802.11a physical and MAC layers for V2V communications. At the physical layer, the IEEE 802.11p uses orthogonal frequency division multiplexing (OFDM) with a channel bandwidth of 10 MHz. The IEEE 802.11p supports data rates ranging from 3 to 27 Mbps using convolutional coding rates of 1/2, 2/3, or 3/4 and binary phase shift keying (BPSK), quadrature phase shift keying (QPSK), 16-point quadrature amplitude modulation (16-QAM), or 64-QAM modulations.

In our experiments, the SISO and MIMO communication performance of the vehicles and vehicle-following controls were evaluated. In the experiments, the follower vehicle moves in lane 1, while the leader vehicle moves in lane 2, with a high-building environment (with poor radio conditions) assigned to this scenario. The vehicle movement situations are demonstrated in Table 4 and Table 5, respectively. As shown in Table 4, the leader vehicle was set to travel through a high-building environment with a constant speed of 36 km/h and suddenly changed lanes in front of the follower vehicle. The speed of the follower vehicle ranged from 40 km/h to 80 km/h. In this setting, the mobility speed effects on the vehicle-following control via V2V SISO and MIMO communications were assessed.

**Figure 7:**
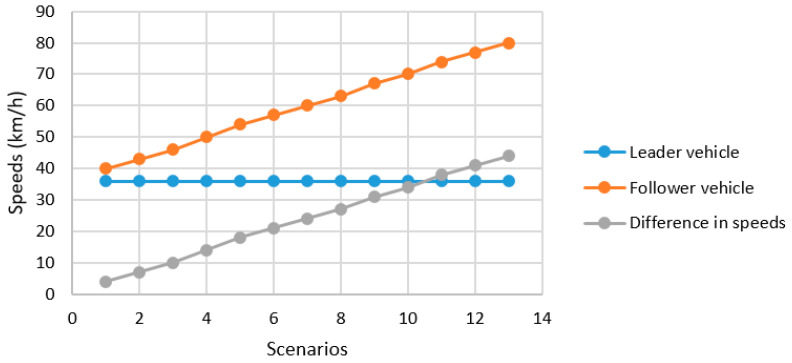
Vehicle speeds from Table 5.

Table 5 and Figure 7 also show the mobility speeds of the follower vehicle, where thirteen test scenarios, with speeds ranging from 40 km/h to 80 km/h, were considered. By varying the follower velocity, the difference in velocity between two vehicles can be from 4 km/h to 44 km/h. Since the mobility speeds are changing, this will affect the wireless communication performance of the SISO and MIMO techniques. Therefore, how well the SISO and MIMO techniques can perform in such situations to support vehicle-following control can be investigated.

Note that in the context of V2V communication, the relative speed between the leader and follower vehicles can result in dispersive channels and Doppler effects. The Doppler effect refers to a change in frequency or wavelength of a radio signal due to the relative motion between the communicating vehicles.

**Figure 8:**
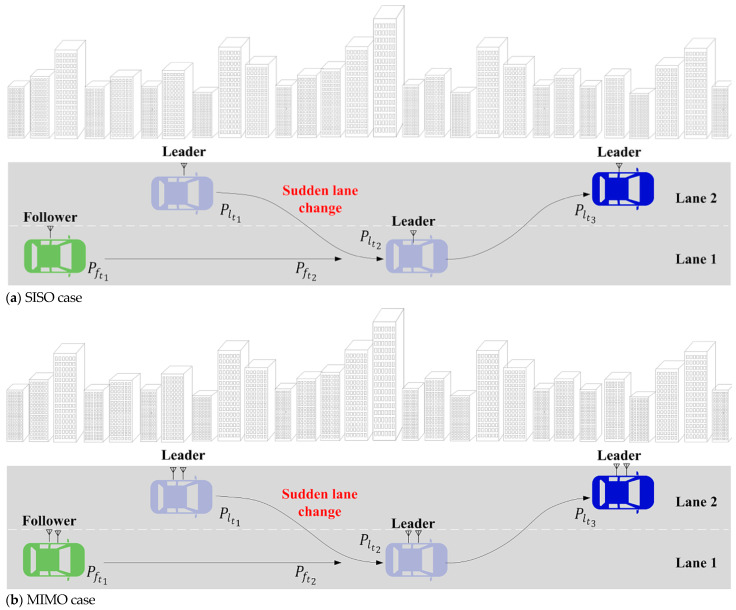
SISO and MIMO cases.

As we mentioned above, the SISO and MIMO communication performance under different vehicle movement speeds will be assessed. The SISO and MIMO test cases are also illustrated in Figure 8, where the figures demonstrate the leader and follower vehicles with their movement direction and patterns, the road lanes (i.e., lanes 1 and 2), the high-building environments, and the SISO and MIMO indications. As in Figure 8a, the leader vehicle has only one antenna and the follower vehicle has one antenna, referring to SISO communication. Since, in Figure 8b, there are two antennas for both the leader and the follower vehicle, MIMO communication is applied for this case.

**Figure 9:**
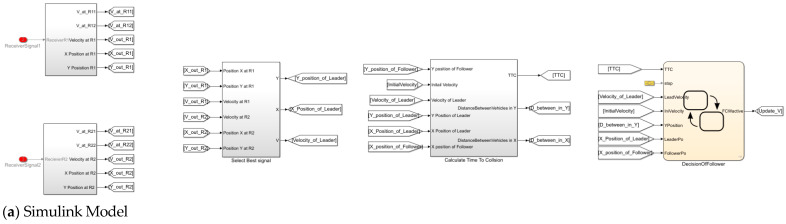

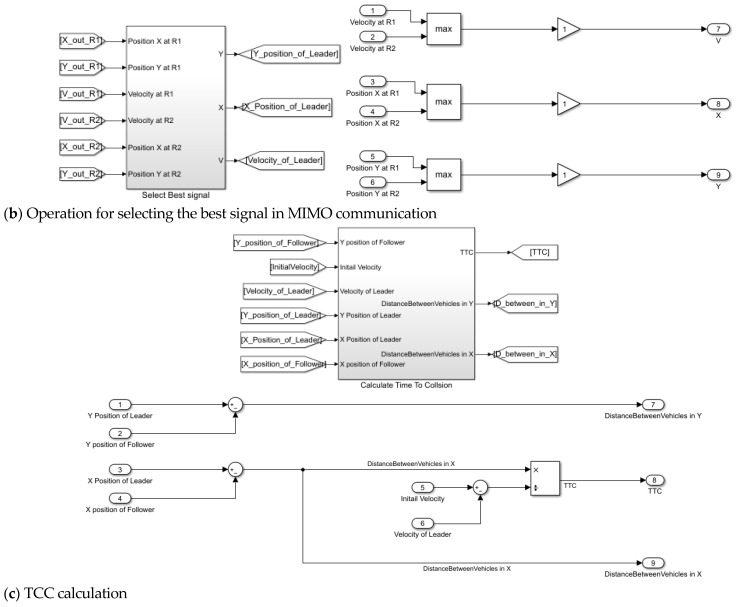
Example of block diagrams implemented in MATLAB/Simulink.

Figure 9 also demonstrates the block diagrams implemented in MathWorks, where the operation for selecting the best signal (for the MIMO communication technique), the TCC calculation, and the decision of the follower are illustrated.

## 4. Results and Discussions

**Figure 10:**
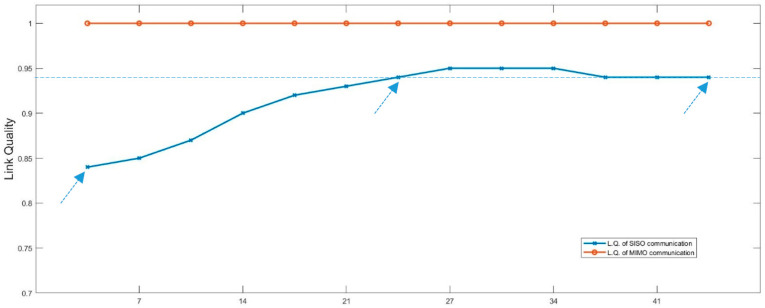
Relationship between link quality (LQ) and the difference in velocity between vehicles (4 to 44 km/h) with SISO and MIMO communications.

Figure 10 shows the relationship between the link quality and the difference in vehicle speeds between the leader and the follower (4 to 44 km/h), where SISO and MIMO communications in an urban environment are applied. The link quality is the ratio of data packets received to data packets sent, measured at the receiver (i.e., the follower vehicle). Thus, 1 indicates a 100% success rate of data transmission via wireless communication. The *y*-axis shows the link quality, and the *x*-axis shows the speed difference between the vehicles. The red colour is the link quality result of the MIMO technique, while the blue colour is the SISO link quality result. The simulation results from our framework demonstrate that the quality of SISO communication is less efficient than MIMO communication. It can be seen that the quality of SISO communication between vehicles with a speed difference of 4 km/h (leader at 36 km/h and follower at 40 km/h) has a communication quality worse than 0.85 and remains worse when compared MIMO communication across the entire range of different vehicle speeds. It was also found that, if the speed of the follower vehicle is increased to 80 km/h, the link quality in the SISO communication is quite good—close to 0.95—due to the decrease in distance between the vehicles, but still worse than MIMO communication. Note that with differences in speed of 24 km/h (leader at 36 km/h and follower at 60 km/h) to 80 km/h, the SISO technique’s performance reaches nearly 0.95. However, for SISO communication, the success rate of data transmission cannot achieve 100%.

According to Section 2.4, if the speed difference ∆v is small, the maximum link lifetime duration $T_{Conn}$ will be large. On the other hand, the bigger the ∆v, the shorter the $T_{Conn}$. Figure 10 shows that with a large difference in velocity between vehicles, like 44 km/h, $T_{Conn}$ will be short. However, MIMO communication achieves a high success rate of data transmission, indicating high communication reliability, although the link lifetime duration is quite small. Since MIMO communication can effectively support strong data transmission in such a situation, this will affect the performance of vehicle control, which will be described next.

**Figure 11:**
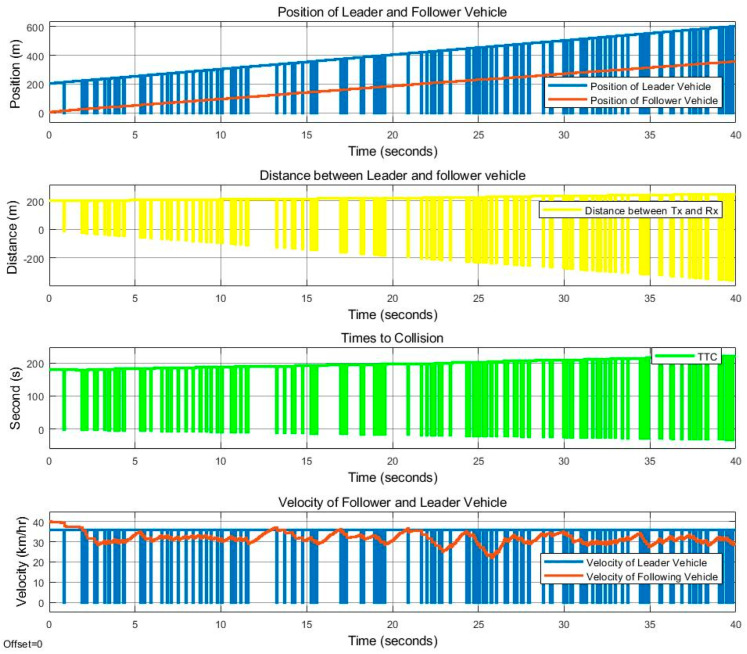
With SISO communication. Position, distance, TTC, and vehicle speed for the time period of 0 to 40 s, at a leader vehicle speed of 36 km/h and a follower vehicle speed of 40 km/h.

**Figure 12:**
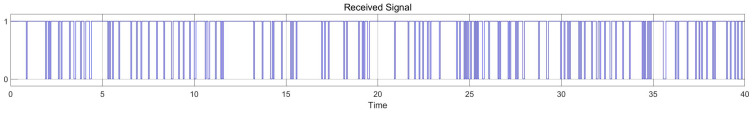
With SISO communication. Received signal at a speed difference of 4 km/h (leader at 36 km/h and follower at 40 km/h). Note that 1 represents success and 0 represents failure.

Figure 11 and Figure 12 show the movement control results of the follower vehicle using SISO communication in an urban environment where the leader vehicle speed is 36 km/h and the follower vehicle speed is 40 km/h, with a speed difference of 4 km/h. Figure 11 shows the positions of both vehicles, where red indicates the position of the follower vehicle, and blue is the position information of the leader vehicle that the follower vehicle received from the SISO communications. Note that the graph shows the positions of the leader and follower as observed by the follower, and when the link with the leader fails, the leader position is plotted as 0. Figure 11 also shows the distance that the follower vehicle can calculate from the received position of the leader vehicle. The TTC, or time-to-collision value, calculated from the distance and the speed of the two vehicles is also presented in the figure. We note that TCC is the ratio of the distance between the vehicles and the difference in velocity between the vehicles. For the speeds of both vehicles in Figure 11, the red line is obtained from the automatic control of the follower vehicle, while blue is the speed of the leader vehicle received via SISO communication. Figure 12 represents the success of the communication between the vehicles, where 1 represents success and 0 represents failure. Figure 12 verifies that there is signal loss at the follower vehicle, as the link quality in this scenario is lower than 0.85, as indicated in Figure 10, causing the follower vehicle to be unable to process the correct value. As a result, the follower vehicle cannot control the distance and speed well.

**Figure 13:**
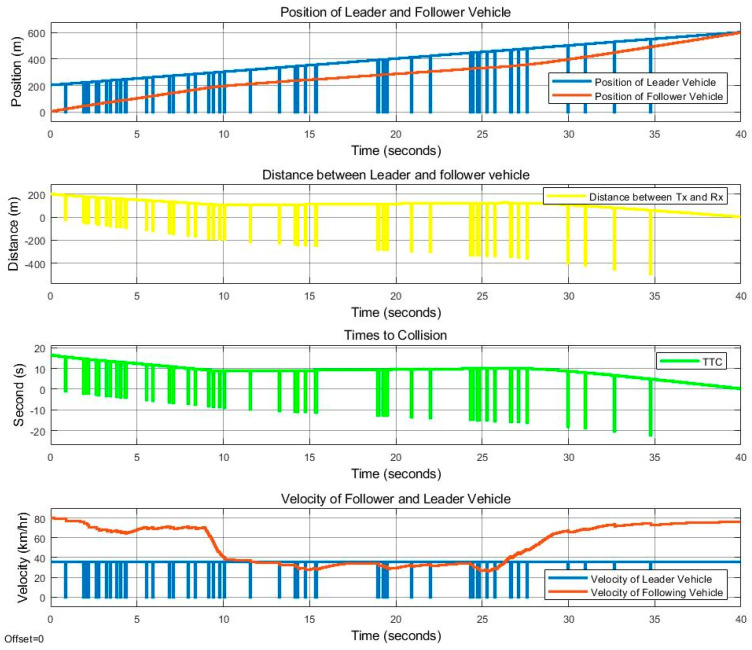
With SISO communication. Position, distance, TTC, and vehicle speed for the time period of 0 to 40 s, at a leader vehicle speed of 36 km/h and a follower vehicle speed of 80 km/h.

**Figure 14:**
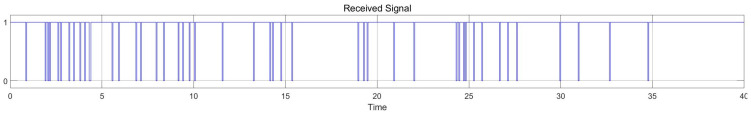
With SISO communication. Received signal at a speed difference of 44 km/h (leader at 36 km/h and follower at 80 km/h).

Figure 13 and Figure 14 show the control movement results of the follower vehicle using SISO communication, where the leader vehicle speed is 36 km/h and the follower vehicle speed is 80 km/h, with a speed difference of 44 km/h. The position of both vehicles, the distance that the follower vehicle calculated from the received position value of the leader vehicle, and the TTC value are provided. It was found that there was still a loss in the signal received by the follower vehicle, similar to the case where the follower vehicle was travelling at a speed of 40 km/h, but to a lesser degree. The link quality in this case was close to 0.95, as shown in Figure 10. At a time period of 0–10 s, it can be seen that the communication quality is still bad. However, it is better than the 40 km/h speed difference case presented in Figure 11. At a time period of 10–25 s, the follower vehicle reduces its velocity to avoid collision with the leader vehicle when the leader vehicle moves with a constant speed of 38 km/h and suddenly changes lanes in front of the follower vehicle. The link quality improves between 25 and 35 s, as shown in Figure 14, as the distance between the two cars decreased. As a result, the control of the follower vehicle can successfully overtake the challenge, but the controllable speed, as shown in the red line in Figure 13 (velocity), still fluctuates.

**Figure 15:**
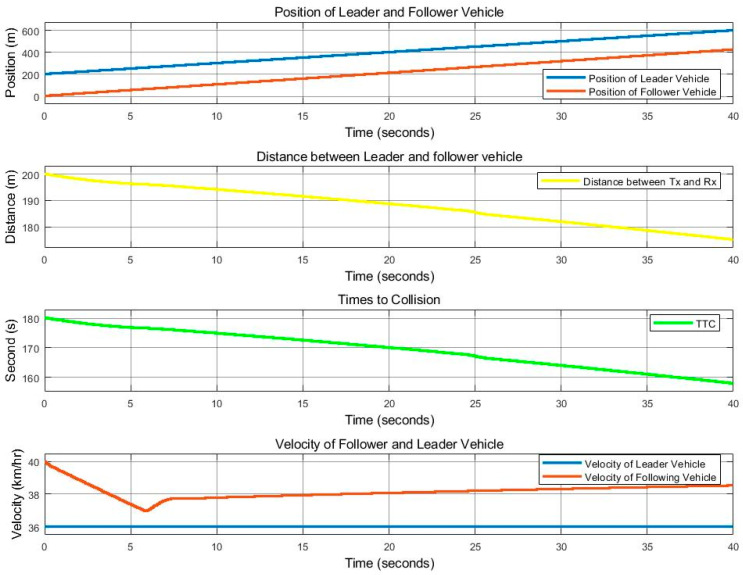
With MIMO communication. Position, distance, TTC, and vehicle speed for the time period of 0 to 40 s, at a leader vehicle speed of 36 km/h and a follower vehicle speed of 40 km/h.

**Figure 16:**
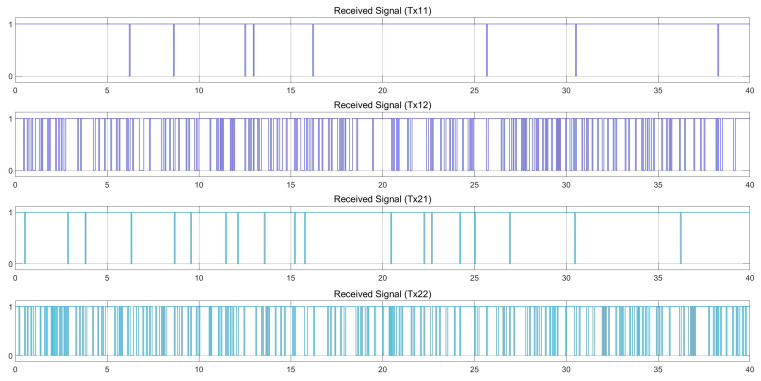
With MIMO communication. Received signal at a speed difference of 4 km/h (leader 36 at km/h and follower at 40 km/h). Tx11, Tx12, Tx21, and Tx22 refer to the Tx to Rx links (e.g., Tx11 → Tx1 to Rx1).

Figure 15 and Figure 16 show the results using MIMO communication, where the leader vehicle speed is 36 km/h and the follower vehicle speed is 40 km/h, with a speed difference of 4 km/h. It was found that there is still a loss in the signal received by the follower vehicle. However, due to the use of MIMO communication and selecting the best signal, as shown in Figure 16, where the signals received at the best channels are chosen for use, the follower vehicle can control its distance and speed better than through SISO communication at the same speed, with the overall link quality approaching 1. We note that Tx11, Tx12, Tx21, and Tx22 refer to the Tx to Rx links (e.g., Tx11 → Tx1 to Rx1).

**Figure 17:**
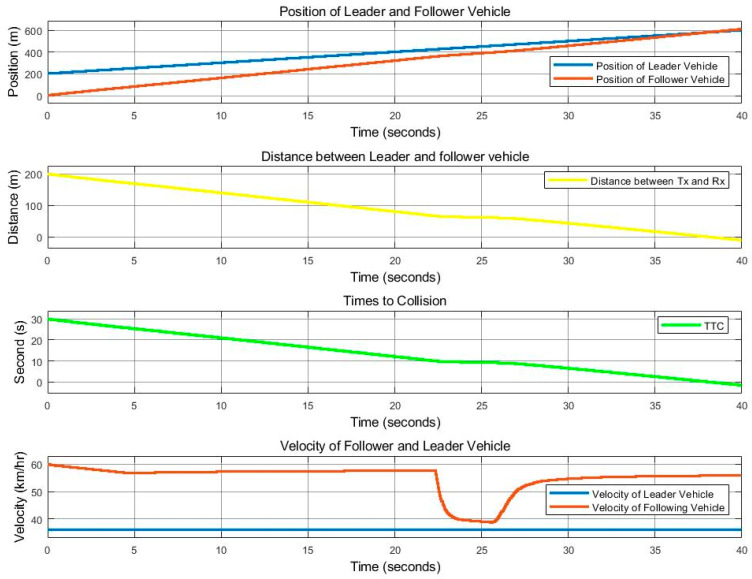
With MIMO communication. Position, distance, TTC, and vehicle speed for the time period of 0 to 40 s, at a leader vehicle speed of 36 km/h and a follower vehicle speed of 60 km/h.

**Figure 18:**
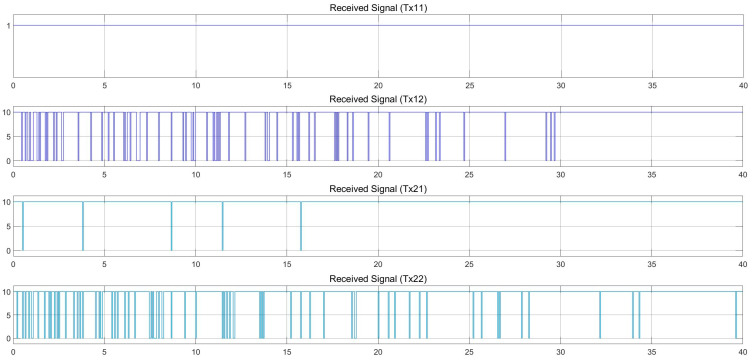
With MIMO communication. Received signal at a speed difference of 24 km/h (leader 36 at km/h and follower at 60 km/h). Tx11, Tx12, Tx21, and Tx22 refer to the Tx to Rx links (e.g., Tx11 → Tx1 to Rx1).

Figure 17 and Figure 18 illustrate the results of using MIMO communication when the speed of the leader vehicle is 36 km/h and the speed of the follower vehicle is 60 km/h, with a speed difference of 24 km/h. Results indicate that there is still a signal loss by the follower vehicle. However, as mentioned before, because the MIMO communication technique was employed, which selected the best signal at each time according to the packets dropped, Figure 18 shows that the follower vehicle can control its distance and speed better than when using SISO communication at the same speed. It was also found that when the speed of the follower vehicle increases, the number of signal errors is reduced, since the distance between two vehicles is quite short and MIMO communication can efficiently support data transmission.

**Figure 19:**
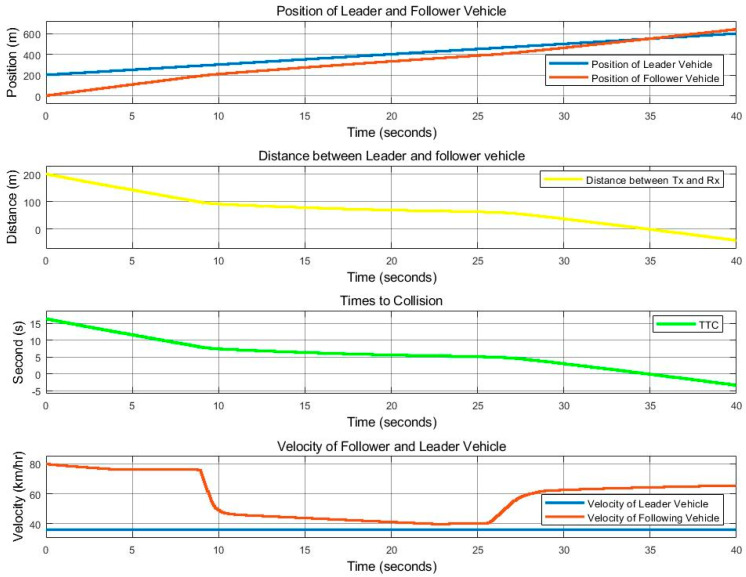
With MIMO communication. Position, distance, TTC, and vehicle speed for the time period of 0 to 40 s, at a leader vehicle speed of 36 km/h and a follower vehicle speed of 80 km/h.

**Figure 20:**
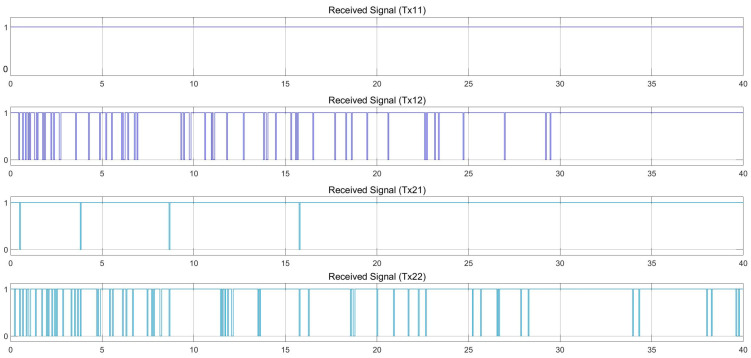
With MIMO communication. Received signal at a speed difference of 44 km/h (leader 36 at km/h and follower at 80 km/h). Tx11, Tx12, Tx21, and Tx22 refer to the Tx to Rx links (e.g., Tx11 → Tx1 to Rx1).

Finally, Figure 19 and Figure 20 demonstrate the MIMO results when the speed of the leader vehicle is 36 km/h and the speed of the follower vehicle is 80 km/h, with a speed difference of 44 km/h. In this case, there is still a signal loss in the communication channel received by the follower vehicle. However, with the MIMO technique selecting the best signal, as shown in Figure 20, the follower vehicle can efficiently control the distance and speed better than when using SISO communication at the same speed. We can observe that when the speed of the follower vehicle increases to 80 km/h, the number of signal errors is reduced more than when the speed of the follower vehicle is set to 40 km/h or 60 km/h. Based on the results presented in this part, findings indicate that the connection quality of SISO communication is less efficient than MIMO communication. As well, MIMO communication has decreased signal loss, allowing the follower vehicle to make accurate decisions while moving.

## 5. Conclusions

In this study, we present a V2V system framework for autonomous vehicle development that allows us to model vehicle functioning, communication channels, and driving scenarios. Experiments with various mobility speeds and SISO and MIMO communication techniques were conducted to investigate communication quality and its impact on vehicle-following control. Experimental results show that SISO communication between vehicles with a speed difference of 4 km/h has a connection quality lower than 0.85. This resulted in unstable control of the follower vehicle speed. The impact of SISO communication can be enhanced by employing MIMO communication technology and selecting the optimal input signal at all times. MIMO communication has decreased signal loss, allowing the follower vehicle to make accurate decisions while moving.

Because this work emphasizes and investigates mobility speed’s effect on vehicle-following control performance via V2V SISO and MIMO communications, more relevant research issues concerning this system should be taken into consideration. In future work, more complicated vehicle movement scenarios with several vehicles and various communication environment effects, like urban environments with dense traffic and highways affected by wireless channels between vehicles, should be taken into account. Finally, the vehicle-following model with an optimal control system solution should be considered.

## Figures and Tables

**Table 1:** A comparison of state-of-the-art technologies.

(a)													
References	Main Study												
[3]	A messaging strategy based on ITS-G5 was developed to send urgent notifications to road users.												
[4]	A VANET, an ITS-G5 and 5G test network, with drone-assisted communication was proposed to improve communication range.												
[5]	Antennas and radio propagation technologies were developed to enhance V2V communications.												
[6]	The evaluation of a cognitive V2V communication system with two active user cooperation.												
[7]	A genetic algorithm for learning the beam shapes of an antenna array was used to improve V2V communications in urban intersections.												
[8]	A federated learning approach for switched beam antennas was used to reduce the latency and throughput overhead in V2V networks.												
[9]	Statistical properties of different taps in different scenarios of a 3D GBSM for V2V MIMO wideband channels were studied.												
[10]	Data exchange between high-speed vehicles in V2X was studied through a MATLAB-based simulation.												
[11]	The OpenStreetMap incorporated with the SUMO simulator and network simulator version 2 were used to investigate the effect of vehicle speeds.												
[12,13]	The effects of several VANET protocols on mobility speed and network density were evaluated.												
[14,15]	The link lifetime between vehicles travelling in the same and opposite directions was compared.												
[16]	The performance impact of mobility speed differences among nodes in a platoon of vehicles was studied.												
[17]	A model-based design framework was developed to model vehicle-following control, autonomous driving scenarios, and communication channels.												
This work	A versatile system-level framework for autonomous vehicle systems was developed. The performance evaluation of the mobility speed effect on vehicle-following control via V2V SISO and MIMO communications was studied.												
(**b**)													
References	V2V communication standard			SISO	MIMO				Simulation	Model-based (MBD)	Model-checking (formal)	Scenario test cases	V2V
	IEEE802.11p	LTE	Other		2 × 2	2 × 4	4 × 4	Other					
[18]	√			√	√		√		√				√
[19]	√								√				√
[20]	√			√	√				√				√
[21]			√		√		√		√				√
[22]		√			√								√
[23]			√					√	√				√
[24]	√			√	√	√	√	√	√				√
[25]	√			√	√				√				√
[26]	√				√				√				√
[27]	√				√	√		√	√				√
[28]		√	√						√				√
[29,30]	√	√							√				√
[31,32,33,34]									√	√	√	√	√
[35]								√	√	√	√	√	√
[36,37]			√						√	√	√	√	√
This work	√			√	√				√	√	√	√ *	√

Note: √ * → Scenario test cases with the effects of mobility speed on autonomous vehicle control.

**Table 2:** Illustrated inputs for the V2V-automated braking system.

Input Values	Description	Range	Unit
Velocity	Vehicle velocity	0–120 km/h	m/s
Acceleration	Vehicle acceleration	−10 m/s^2^ to 10 m/s^2^	m/s^2^
Throttle state	Vehicle throttle state	0–100	%
Brake state	Brake state	0–100	%
Steering angle	Steering wheel rotation angle	±180	degree
Target ID	Vehicle ID	2–4 vehicles	number of vehicles
Range (R)	Distance between vehicles	20–40 m	m

**Table 3:** Parameters of the IEEE 802.11p and the IEEE 802.11a.

Parameters	802.11a	802.11p	Difference
Bit rate (Mbit/s)	6, 9, 12, 18, 24, 36, 48, 54	3, 4.5, 6, 9, 12, 18, 24, 27	Half
Modulation Mode	BPSK, QPSK, 16-QAM, 64-QAM	BPSK, QPSK, 16-QAM, 64-QAM	No changes
Code rate	1/2, 2/3, 3/4	1/2, 2/3, 3/4	No changes
Number of subcarriers	52	52	No changes
Symbol duration (µs)	4	8	Double
Guard time (µs)	0.8	1.6	Double
Preamble duration (µs)	16	32	Double
Subcarrier spacing (MHz)	0.3125	0.15625	Half

**Table 4:** The vehicle movement situations.

Vehicle	Velocity of Each Vehicle with Each Communication Technique		Environment	Leader Lane Change
	SISO	MIMO		
Leader	36 km/h	36 km/h	High-building	Suddenly
Follower	40–80 km/h	40–80 km/h	High-building	No

**Table 5:** Vehicles’ movement speeds in each test scenario.

Scenarios	Leader Velocity	Follower Velocity	Difference in Velocity Between Vehicles
1	36 km/h	40 km/h	4 km/h
2	36 km/h	43 km/h	7 km/h
3	36 km/h	46 km/h	10 km/h
4	36 km/h	50 km/h	14 km/h
5	36 km/h	54 km/h	18 km/h
6	36 km/h	57 km/h	21 km/h
7	36 km/h	60 km/h	24 km/h
8	36 km/h	63 km/h	27 km/h
9	36 km/h	67 km/h	31 km/h
10	36 km/h	70 km/h	34 km/h
11	36 km/h	74 km/h	38 km/h
12	36 km/h	77 km/h	41 km/h
13	36 km/h	80 km/h	44 km/h

**Figure A1:**
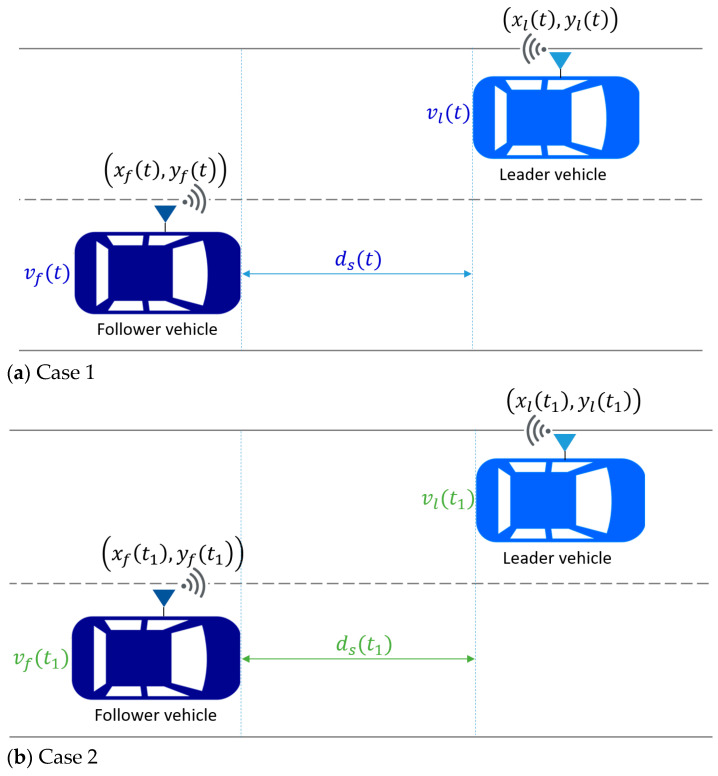

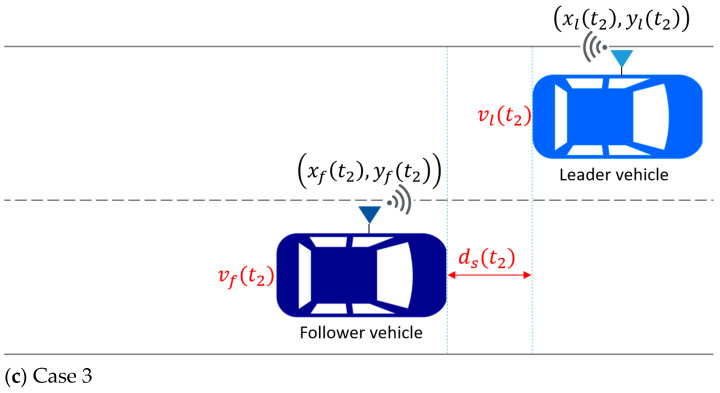
An illustration of vehicle movements on a straight road and the TCC calculation.

## Data Availability

The data used in the current study are available by the authors upon request.

## Appendix A. TTC Calculation

The TTC is the ratio of the distance and velocity between vehicles (i.e., TTC(t) in (A1)). It is expressed in Figure A1, where $v_{f}(t)$ is the follower’s velocity relative to the velocity of the leader vehicle $v_{l}(t)$, and $d_{s}(t)$ is the distance between both vehicles. The distance can be calculated from the vehicle positions (i.e., $\left(x_{f}(t),y_{f}(t)\right)$ and $\left(x_{l}(t),y_{l}(t)\right)$) provided by the GPS module, where latitudes and longitudes from the GPS are transformed to x and y values based on their position in the tangential plane.

(A1)
$$TTC\left(t\right)=\left(\frac{d_{s}\left(t\right)}{v_{f}\left(t\right)-v_{l}\left(t\right)}\right)$$
